# Ankle fusion with tibiotalocalcaneal retrograde nail for fragility ankle fractures: outcomes at a major trauma centre

**DOI:** 10.1007/s00590-021-03171-1

**Published:** 2021-11-24

**Authors:** Victor Lu, Maria Tennyson, James Zhang, Azeem Thahir, Andrew Zhou, Matija Krkovic

**Affiliations:** 1 School of Clinical Medicine, University of Cambridge, Cambridge, CB2 0SP UK; 2 Department of Trauma and Orthopaedics, Addenbrooke’s Hospital, Cambridge, CB2 0QQ UK; 3 Christ’s College, St. Andrew’s Street, Cambridge, CB2 3BU UK

**Keywords:** Ankle fusion, Tibiotalocalcaneal nailing, Ankle fractures, Mobility, Co-morbidity

## Abstract

**Purpose:**

Fragility ankles fractures in the geriatric population are challenging to manage, due to fracture instability, soft tissue compromise, and patient co-morbidities. Traditional management options include open reduction internal fixation, or conservative treatment, both of which are fraught with high complication rates. We aimed to present functional outcomes of elderly patients with fragility ankle fractures treated with retrograde ankle fusion nails.

**Methods:**

A retrospective observational study was performed on patients who underwent intramedullary nailing with a tibiotalocalcaneal nail. Twenty patients met the inclusion criteria of being over sixty and having multiple co-morbidities. Patient demographics, AO/OTA fracture classification, intra-operative and post-operative complications, time to mobilisation and union, AOFAS and Olerud-Molander scores, and patient mobility were recorded.

**Results:**

There were seven males and thirteen females, with a mean age of 77.82 years old, five of whom are type 2 diabetics. Thirteen patients returned to their pre-operative mobility state, and the average Charlson Co-morbidity Index (CCI) was 5.05. Patients with a low CCI are more likely to return to pre-operative mobility status (*p* = 0.16; OR = 4.00). All patients achieved radiographical union, taking on average between 92.5 days and 144.6 days. The mean post-operative AOFAS and Olerud-Molander scores were 53.0 and 50.9, respectively. There were four cases of superficial infection, four cases of broken or loose distal locking screws. There were no deep infections, periprosthetic fractures, nail breakages, or non-unions.

**Conclusion:**

Tibiotalocalcaneal nailing is an effective and safe option for managing unstable ankle fractures in the elderly. This technique leads to lower complication rates and earlier mobilisation than traditional fixation methods.

## Introduction

Fragility ankle fractures are increasing in incidence, and is now the third most common type of fracture in the elderly, after hip and distal radius fractures, with 184 cases per 100,000 people per year [1]. The rising life expectancy contributes to the growing number of cases, which has been projected to increase 25% by 2050 [1, 2]. Ankle fractures show a bimodal age distribution, with the majority in men seen between the ages of 15 and 24, whilst the highest incidence in females is between 75 and 84 years old [2]. These fractures are difficult to manage not least because of osteoporosis, whose incidence is also on the rise due to increasing life expectancy, as well as other co-morbidities such as diabetes [3, 4]. Osteoporosis is responsible for over nine million fractures a year, and creates fractures patterns that are more complex and unstable [5]. Despite a Korean study suggesting that body mass index (BMI) rather than bone mineral density (BMD) is a risk factor for ankle fracture, which could be due to the small sample size and retrospective nature of the study [6], a meta-analysis of over 25,000 patients demonstrated a significant association between fragility ankle fractures in the elderly population and reduction in BMD [7].

A fragility ankle fracture was defined as one that occurs in patients over 60, as a result of minimal trauma, and in patients with osteoporotic bone [8, 9]. A meta-analysis with over 60,000 patients concluded that a previous fragility fracture (FF) located anywhere increases the risk of acquiring a subsequent fragility fracture (RR = 1.86; 95% CI = 1.75–1.98) which is largely independent of BMD [10]. Nevertheless, some studies suggest that ankle FFs have a lower predictive value for subsequent FFs than FFs occurring at more typical osteoporotic locations such as the hip and vertebrae [11]. This could be because ankle fractures have a weaker dependence on age and bone mass [12], are more driven by mechanical factors such as twisting or distortion rather than osteoporosis [13], and have a stronger relationship with lifestyle factors [14]. Risk factors also differ from FFs at other sites, for example menopause was strongly and linearly related to wrist fractures but not to ankle fractures [15]. Multivariate analyses of a cohort of patients aged ≥ 50 concluded that those with ankle FFs who are still physically active or at low/moderate risk according to the WHO’s fracture risk assessment tool (FRAX) may not need subsequent investigation or treatment [13].

The goal for ankle fracture primary management includes anatomical restoration of the tibiotalar mortise, a stable and pain-free ankle, and a rapid return to baseline mobility. Particularly in the elderly, important considerations are early mobilisation and weight-bearing, the benefits of which have been shown in a study investigating mortality and immobility in hip fracture patients [16]. Prolonged periods of non-weight bearing is difficult for the elderly, may lead to complications such as pressure ulcers and deep vein thrombosis, and often leads to a lengthy stay at a nursing home. Management of fragility ankle fractures in the elderly is difficult, because of poor bone quality, healing ability, soft tissue condition, suboptimal skin quality, and lack of patient compliance. Intramedullary nails are beneficial since they allow early weight-bearing, require only a small incision, and minimises soft tissue trauma [17]. Since 2005, the literature contains optimistic reports of using tibiotalocalcaneal (TTC) nails for treatment, with no non-union [18] and immediate weight-bearing post-operatively [19]. However, few studies assessed functional outcomes with adequate follow-up times [18–22].

This paper presents a cohort of fragility ankle fractures in the elderly, treated with retrograde ankle fusion nails. The primary objective was to assess whether the number of co-morbidities is associated with a return to pre-operative mobility status and post-operative complications. The secondary objective was to assess the time to radiographical union and patient reported outcome measures (PROMs).

## Methodology

The patient record database was retrospectively reviewed for patients who received a retrograde ankle fusion nail. Our inclusion criteria were:

- Age over 60.
- Patients who are able to give informed consent.
- Patients with two or more co-morbidities.
- Patients who are unable to comply with post-operative non-weight bearing instructions due to mental or physical reasons.
- Patients with poor bone stock, verified by radiological evidence of osteopenia or a history of fragility fractures.
- Unstable fracture pattern necessitating operative management, as determined by a medial clear space ≥ 5 mm on antero-posterior radiographs taken in dorsiflexion.
- Poor soft tissue condition around the ankle upon physical examination

In addition to excluding patients who did not meet the aforementioned criteria, the following patients were also excluded:

- Patients who were not fit for anaesthesia
- Patients with high-energy mechanism of injury
- Patients with peripheral vascular disease
- Patients with previous fracture of the affected limb
- Pathological fractures

Out of 171 patients who received a hindfoot nail, twenty patients met the inclusion criteria. Thirteen were female and seven were male. The mean age was 77.8 years old (range 61 to 95). Injury was low energy in all patients, including the nine patients who had open fractures, two of whom had Gustilo-Anderson type 3a fractures and one with Gustilo-Anderson type 3b fracture, all of whom required soft tissue coverage. Fractures were classified using the Arbeitsgemeinschaft für Osteosynthesefragen/Orthopaedic Trauma Association (AO/OTA) classification. There were seven trimalleolar fractures (three AO/OTA 44C1, three 44B2, one 44B1), 12 bimalleolar fractures (six AO/OTA 44C1, four 44A2, two 44B2), and one pilon fracture (AO/OTA 43C1). Twelve operations were performed as primary fracture management, four for failed conservative treatment, three for failed open reduction internal fixation (ORIF), and one for failed TTC nailing at a different hospital. All ankle fusion procedures were performed by one consultant orthopaedic surgeon (MK). The average follow-up time was 499.3 days.

All patients had multiple co-morbidities, which was quantified using the Charlson co-morbidity index (CCI) [23]; average score was 5.05. CCI produces a co-morbidity-age combined risk score, and can be converted using a formula to give a predicted 10-year survival percentage, based on the 10-year survival from a theoretical low-risk population (98.3%).

$$ {\text{Predicted}}\;10{\text{-year}}\;{\text{survival}} = 0.983^{{(e^{{0.9\; \times \;CCI}} )}}  $$

As CCI increases from 0 to 6, predicted 10-year survival percentage drops as follows: 99, 96, 90, 77, 53, 21. We defined a high CCI as a value that has a corresponding 10-year survival of less than 50%, i.e. CCI ≥ 5; the remainder (CCI < 5) is defined as low CCI.

The decision to proceed with TTC nailing was made by the consultant, following assessment of patient’s pre-operative mobility, co-morbidities, bone quality, fracture pattern/stability, and ability to comply with non-weight bearing status. On admission, patients were managed with our standard trauma protocol. All open fracture patients received prophylactic antibiotics, as per BOAST guidelines for open fracture management [24]. A standard ankle fusion procedure was followed. Three operations were performed by senior fellows, the rest (85%) were consultant-led. Patients on average spent 10.8 days in hospital. One patient spent 31 days due to a heel ulcer which got infected, leading to osteomyelitis of the calcaneum. This was successfully treated with teicoplanin and ciprofloxacin. PROMs were collected twelve months after surgery, namely AOFAS ankle-hindfoot score and Olerud and Molander (O&M) score. PROMs were unable to be collected in three patients who passed away within one month of surgery.

Statistical analysis was performed using IBM SPSS Statistics version 27. Categorical binary data was analysed with Pearson’s Chi-squared test. A significance value of *p* ≤ 0.05 was used.

This study was registered with the clinical research department on May 25th 2021; registration number PRN9832.

## Results

Demographical information is provided in Table 1 and clinical outcomes in Table 2. Thirteen patients (65%) returned to their pre-operative mobility state and seven patients had inferior post-operative mobility compared to pre-operative mobility. Five patients who could previously walk independently subsequently required a crutch at all times. Two patients who previously used a walking frame subsequently required a wheelchair. Compared to those with a high CCI score (CCI ≥ 5), patients with a low CCI score (CCI < 5) were more likely to return to their pre-operative mobility status (*p* = 0.16; OR = 4.00).

**Table 1:** Patient demographics

Total population	20
Male	7 (35%)
Female	13 (65%)
Age (years)	77.82 (61–95)
Male	70.71 (61–95)
Female	82.8 (66–89)
BMI	30.1 (16.65–49.54)
Smoking status	
Ex-smoker	10 (50%)
Non-smoker	8 (40%)
Current smoker	2 (10%)
Diabetes Mellitus	
Yes	5 (25%)
No	15 (75%)
Charlson Comorbidity Index	5.05 (3–9)
ASA Grade	2.44 (2–4)
*Fracture Pattern*	
Bimalleolar	12 (60%)
Trimalleolar	7 (35%)
Pilon	1 (5%)
*Fracture Type*	
Closed	11 (55%)
Open Gustilo-Anderson 2	6 (30%)
Open Gustilo-Anderson 3a	2 (10%)
Open Gustilo-Anderson 3b	1 (5%)
*Fracture Classification*	
AO/OTA 43C1	1 (5%)
AO/OTA 44A2	4 (20%)
AO/OTA 44B1	1 (5%)
AO/OTA 44B2	5 (25%)
AO/OTA 44C1	9 (45%)
*Pre-Injury mobility*	
Walk independently	5 (25%)
Crutches	7 (35%)
Frame	8 (40%)

**Table 2:** Clinical outcomes

Time to mobilisation (days)	7.6 (2–24)
Bone union time interval (days)	92.5 to 144.6
Hospital length of stay (days)	10.8 (2–31)
Average operative time (min)	131.2 (68–227)
Average follow-up time (days)	499.3 (51–1360)
Post-operative complications	8 (40%)
Deaths within 6 months	3 (15%)
AOFAS score 6 months after operation	53.0 (17–88)
Olerud-Molander score 6 months after operation	50.9 (20–85)
*Mobility 12 months after operation*	
Walk independently	0
Crutches	6 (30%)
Frame	12 (60%)
Wheelchair	2 (10%)

After 24 h of strict elevation of the affected limb, all patients were allowed to fully mobilise, as far as pain could be tolerated. The average time to mobilisation was 7.63 days. Those who could not mobilise after day 4, all suffered from complications. One patient who could only mobilise after 24 days had a hindfoot ulcer, grade 3 pressure ulcers, and severe back pain due to non-union of a previous public rami fracture.

The average length of hospital stay was 10.8 days (range 2–31). Patients were re-evaluated clinically and radiography on average 30 days after discharge, followed by clinics at 4 week intervals. All patients eventually achieved radiographic union, defined as the presence of bony bridging on antero-posterior and lateral X-ray views, together with painless full weight-bearing. Bone union took on average between 92.5 and 144.6 days to occur. Average AOFAS score 6 months post-injury was 53.0 (17–88). The O&M score was not calculated pre-injury, but the average value 6 months post-injury was 50.9 (range 20–85).

The six-month mortality rate was 15% (3/20). One patient passed away eleven days after surgery, due to post-operative ileus, causing vomiting and aspiration pneumonia, eventually leading to respiratory failure. Surgical complications included four superficial infections (20%), treated with topical antibiotics. Patients with a high CCI were more likely to acquire superficial infections (*p* = 0.264, OR = 3.857). Four patients experienced pain due to broken or loose distal locking screws, which were subsequently removed. Otherwise, metalwork removal was not performed. One patient experienced paraesthesia in the distribution of superficial peroneal nerve and sural nerve, probably damaged iatrogenically, or due to scarring of soft tissue. There were no deep infections, periprosthetic fractures, nail breakages, or non-unions. However, one patient had delayed union (279 days to union), which eventually united after regular observation, and lymphoedema causing an equinus deformity, leading to a low AOFAS score of seventeen.

## Discussion

### Patient demographics and surgical management

Surgical management of fragility fractures in the elderly is challenging, with traditional management yielding poor results [25]. Conservative management using fracture manipulation or plaster mobilisation produced a non-union rate of 73% [26], with 79% experiencing chronic pain [27]. ORIF produces poor outcomes in the elderly due to patient-specific conditions such as poor condition of the skin and soft tissue, which is exacerbated by the fracture, poor bone quality, limited pre-injury mobility, advanced age, complex, and unstable fracture patterns [28]. Surgical wound complications are a concern, as well as an increased risk of deep infection and delayed wound healing due to conditions such as diabetes and peripheral vascular disease, and potential use of corticosteroids [29]. Studies that performed ORIF reported a 19% non-union rate and 43% (37/86) patient dissatisfaction rate [25]. Beauchamp et al. reported a 23% wound complication incidence, and anatomical fixation was achieved in only 54% (38/71); this was significantly more biased towards men (17/22 in men versus 21/49 in women), perhaps due to an increased proportion of osteoporotic bone in women during surgery, increasing the morbidity associated with ORIF [4]. Georgiannos et al. performed a randomised control study between patients treated with TTC nailing and ORIF; the former cohort had a reoperation rate of 2.7%, whilst the latter was 13.8% [3]. Ali et al. reported optimistic results using ORIF, with 8.7% (10/115) malunion rate and only one patient with non-union [30]. However, it is a biased study since they excluded those with severe mobility problems, who are most likely to fall and injure themselves, and likely to have osteoporotic bones. Litchfield et al. suggested that inactivity mitigates against ORIF success, and those who were active on their feet pre-injury had the best results [25]. However, the majority of our cohort were dependent on a crutch or frame pre-injury, and all had some degree of impaired mobility.

The average age of our cohort at time of injury was 77.8, which is similar to other cohorts [18, 21]. According to our definition for a fragility fracture, only those over 60 years old were included; this itself was suggested to be a negative prognostic factor for surgical treatment of trimalleolar ankle fractures according to O&M score (*p* = 0.000002) and VAS score (*p* = 0.048) [31]. The average age of women and men in our cohort were 82.8 and 70.7, respectively; this was surprising given that women over 50 have a fourfold higher rate of osteoporosis than men [32]. The average hospital stay of 10.8 days was shorter than cohorts treated conservatively or by ORIF [27, 28].

The proportion of open fractures in our cohort (45%) is higher than those in the literature [8, 22], whilst some cohorts had no patients with open fractures [18, 19]. This could be due to our clinic being located in a major trauma centre. Although commonly associated with high-energy injuries, all nine open fracture patients in our cohort acquired low-velocity trauma, with six suffering a fall from standing height, and three tripping over on stairs. The open fracture was likely caused by sharp fracture fragments piercing through the soft tissue and skin in patients with poor soft tissue condition. A large proportion of our cohort (40%) received TTC nailing as a ‘salvage procedure’ after unsuccessful prior management. Despite being higher than other cohorts [21], a fair comparison may not be possible, due to the lack of definitive guidelines for when TTC nailing should be used first-line, with surgeons themselves deciding if patients fit the criteria to receive TTC nailing. Nevertheless, this salvage technique has shown promising results in previous reports, with radiological union occurring three months following surgery [33].

**Fig. 1:**
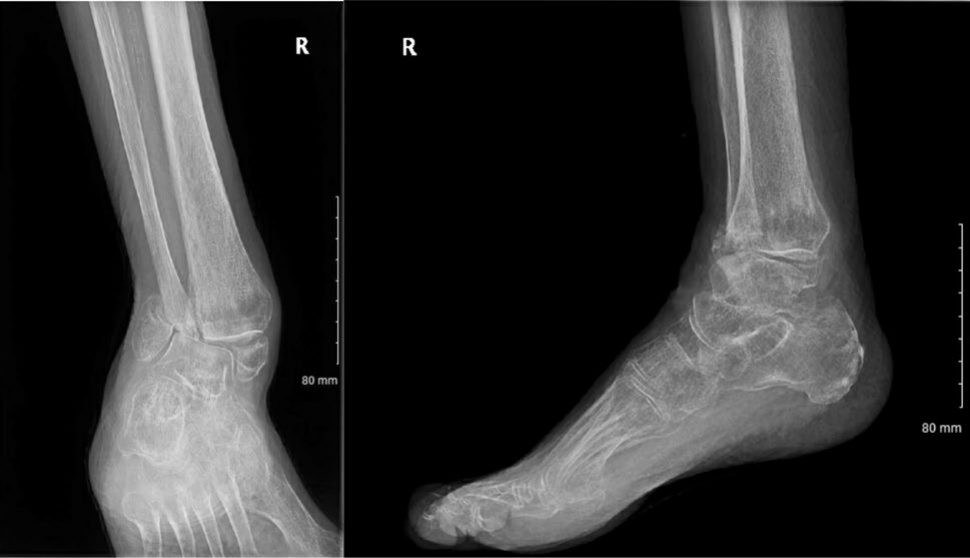
*Left*—AP view before nail insertion in a patient whose joint was prepared; *Right*—Lateral view before nail insertion in a patient whose joint was prepared

**Fig. 2:**
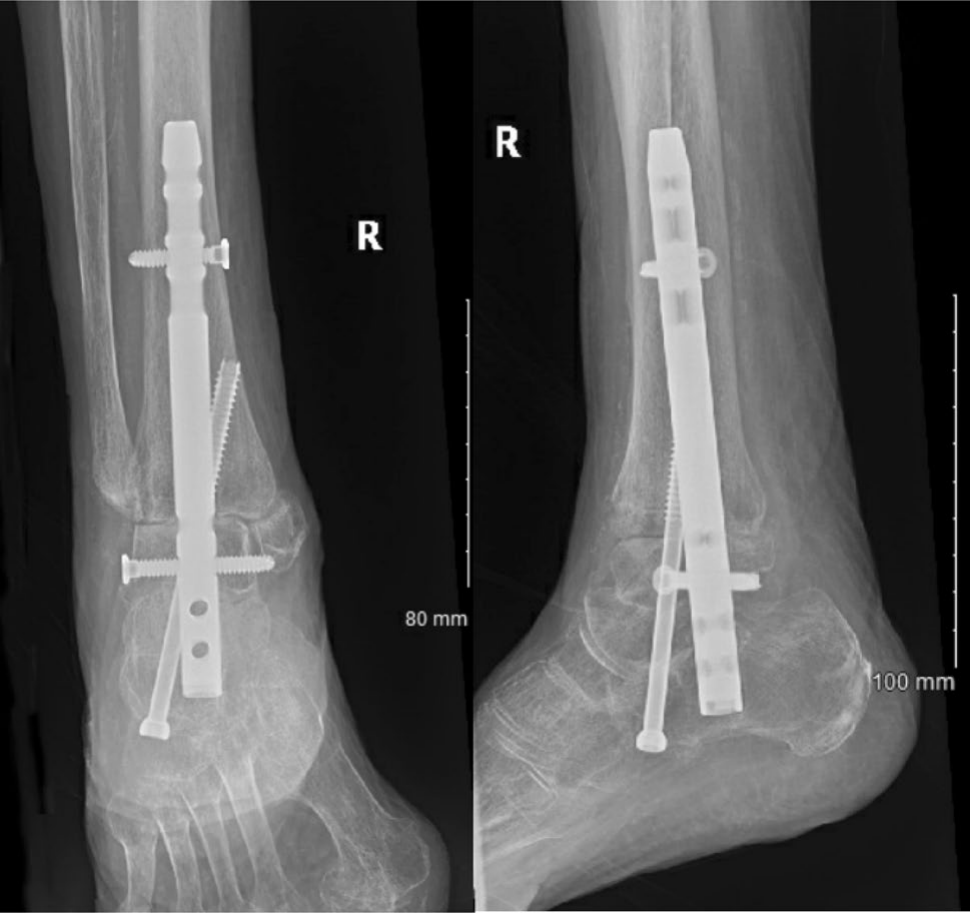
*Left*—AP view after nail insertion with joint preparation; *Right*—Lateral view after nail insertion with joint preparation

**Fig. 3:**
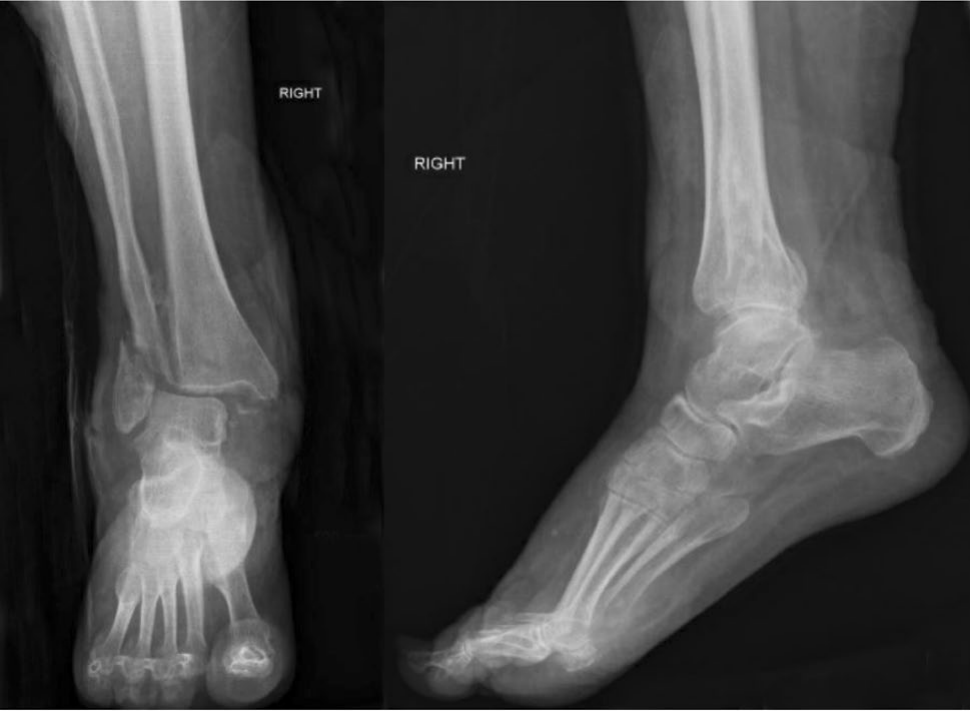
*Left*—AP view before nail insertion in a patient whose joint was not prepared; *Right*—Lateral view before nail insertion in a patient whose joint was not prepared

**Fig. 4:**
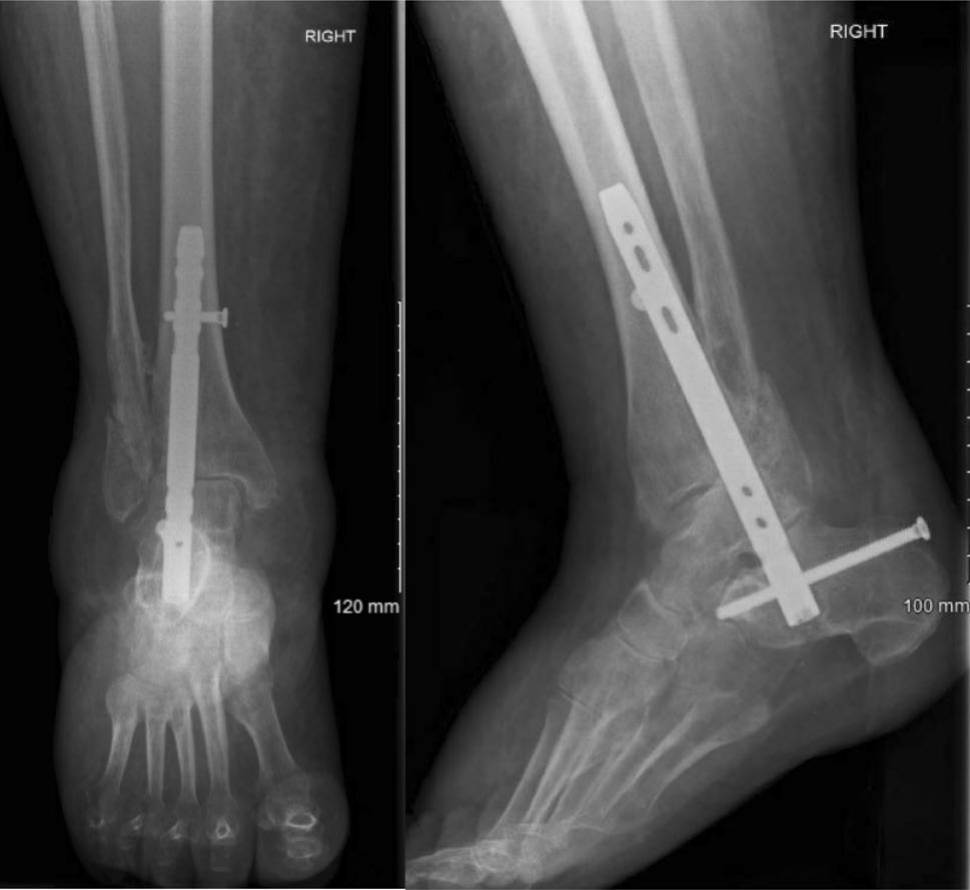
*Left*—AP view after nail insertion with joint not prepared; *Right*—Lateral view after nail insertion with joint not prepared

Ankle and subtalar joints were prepared prior to nail insertion, meaning that they were denuded of cartilage down to subchondral bone (Figs. 1, 2). Surgeons in three operations elected to not prepare the joints (Figs. 3, 4). Studies mention that preparing the subtalar joint reduces non-union rate [20, 34]; nevertheless, whether or not subtalar joint needs open debridement remains a contentious point. The surgeons who elected to not prepare the joints felt that doing so would devascularise the talar fragments excessively, increase surgical insult and create an unnecessarily invasive procedure that would introduce wound healing issues, in return for arthrodesis union which is hard to achieve in a host with multiple co-morbidities. Preparing the joint is not a common routine in the literature, and all three patients managed without joint preparation achieved radiographical union. Perhaps joint preparation is more important for young, active patients, whereby hardware failure is more likely due to cyclic loading on the metalwork.

### Mobility

Ankle fusion is not necessarily a life-changing procedure. Georgiannos et al. reported that 81.8% of patients treated with a hindfoot nail returned to their pre-operative mobility status [3]. The figure was 65% in our cohort. We found that having a low number of co-morbidities (low CCI score) is positively correlated with regaining one’s pre-operative mobility (*p* = 0.160; OR = 4.00). However, the association was not statistically significant, probably because our sample size was too small to be adequately powered. Additionally, we noticed that those who took longer than 4 days post-surgery to mobilise all suffered some form of post-operative complication. This is similar to Lemon et al. who found that all who failed to mobilise within 72 h of surgery suffered a post-operative medical complication [19]. Furthermore, we found that patients with a high CCI score are likely to get superficial infections (*p* = 0.264; OR = 3.857). All this suggests that patients who have more co-morbidities (higher CCI score), who are less likely to return to pre-operative mobility status, are also expected to suffer from post-operative complications, likely superficial infections.

### Diabetes mellitus

Five patients (25%) in our cohort were type 2 diabetics, two of whom had superficial wound infection (40%). Management of patients with diabetes adds another layer of complexity, given the high infection rates and wound healing problems that are known to be associated [35]. In their cohort of 93 patients treated with ORIF, Low and Tan [36] reported five patients with wound infections, all of whom were type 2 diabetics. With a 50% infection rate in their diabetic cohort, this illustrates that infection is a serious problem in surgically treated diabetics with ankle fractures. Diabetics are also at increased risk of other complications such as non-union and post-traumatic arthritis; Blotter et al. suggested a 2.76-fold higher relative risk for post-operative complications in patients with diabetes mellitus compared to control group [37]. This could be due to diabetic neuropathy, leading to unprotected weight-bearing on the senseless foot. Furthermore, diabetes and obesity are closely interlinked, with four out of five diabetics (80%) in our cohort being obese (BMI ≥ 30). This could be due to biochemical relationships between insulin signalling and adipose tissue, including inhibition of intracellular lipase and increased triacylglycerol synthesis in liver [38]. Not only is average BMI in ankle fracture patients higher than age-matched controls [6], but a retrospective study of 48 patients suggested that morbidly obese patients (BMI ≥ 40) is a negative prognostic factor for ankle fracture management [31].

### Complications

Our overall complication rate was 20% (4/20). This falls within the range of 18–22.6% for TTC nailing as quoted in a recent systematic review [17], which also reported that fibula nails have a lower complication rate of 0–22%. Nevertheless, our complication rate was lower than patient cohorts treated with ORIF [3, 4]. Perhaps due to our high percentage of open fractures, superficial infection rate was higher than the range of 0–6.5% quoted in the literature [8, 22]. We report no cases of deep infection or non-unions, which is very favourable compared to other management options such as ORIF [4, 27], as well as other studies utilising TTC nailing [8, 20, 22], with non-union rates of 30% being reported [20]. We also report no periprosthetic fractures, even though a long nail was not used, which was suggested to prevent periprosthetic fractures [22]. Perhaps this was due to low functional demands in our cohort. Four patients required removal of a broken or loose locking screw; one used a crutch whilst three did not need a walking aid. These patients may have been too ‘active’ to receive TTC nailing; however, they were selected due to their poor skin and soft tissue conditions and perceived lack of ability to comply with non-weight bearing instructions.

### PROMs and bone union

AOFAS scores was reported in only one other study [39]. Our average value of 53.0 is lower than their value of 85.4. Reason could be twofold. We reported AOFAS scores at 6 months post-injury, but Al-Ashhab et al. reported it at final follow-up [39]. Also, 45% of our patients had open fractures, whereas their cohort had no open fractures [39]. Our average O&M score 6 months post-injury of 50.9 concurs with a recent systematic review on intramedullary nailing in ankle fractures, which suggested that the mean O&M score for patient cohorts treated with TTC nailing in the literature was 50–62 [17]. Nevertheless, the limitations of the systematic review, namely the differences in patients included, varying definitions of outcome scores, and the low quality of included studies precluded the ability to draw definitive conclusions [17].

Few studies report time to union. Bone union took on average 92.5 days to 144.6 days to occur, which was longer than the average time to union of 63 days reported by Jonas et al. [18]. However, this may not be a fair comparison since in retrospective studies, finding when exactly union occurred is difficult. Patients do not have frequent, evenly-spaced radiological follow-ups. Furthermore, COVID-19 has exacerbated this issue, with virtual follow-ups prolonging the gap between radiological checks. This is the reason we preferred to give a time interval within which bone union occurs, rather than a definitive number.

### Limitations

Our study has a few limitations, not least being the retrospective study design. There was no control group to compare with other management options such as ORIF or conservative treatment. Our population size is relative small and heterogeneous, with patients having various fracture patterns and classifications. Also, we could not obtain pre-operative O&M scores, which would have been useful to compare with post-operative scores. Our cohort included patients who received TTC nailing as a primary treatment, as well as patients who received TTC nailing as a ‘salvage procedure’ after failed treatment using more conventional methods. Our population size was not large enough to provide a comparison between these two groups. Furthermore, there is a lack of definitive guidelines for when TTC nailing should utilised as primary management for ankle fractures.

## Conclusion

TTC nailing is an effective treatment methodology for the low-demand geriatric patient with unstable fragility ankle fractures, and should be added to the armamentarium of management options for fragility ankle fractures in the elderly. It effectively stabilises the hindfoot and encourages early weight-bearing, maintaining mobility, which is important for preventing the loss of socio-economic independence. TTC nailing limits soft tissue injury and has few complications, compared to other treatment options such as ORIF or conservative management. It is not a life-changing procedure, with many being able to return to their pre-operative mobility status, however, the number of co-morbidities is a negative predictive factor for returning to pre-operative mobility status, and a positive predictive factor for the development of post-operative complications.

## Declarations

### Data availability

The authors confirm that the data supporting the findings of this study are available within the article [and/or] its supplementary materials.
